# Metformin may adversely affect orthostatic blood pressure recovery in patients with type 2 diabetes: substudy from the placebo-controlled Copenhagen Insulin and Metformin Therapy (CIMT) trial

**DOI:** 10.1186/s12933-020-01131-3

**Published:** 2020-09-26

**Authors:** Christian Stevns Hansen, Louise Lundby-Christiansen, Lise Tarnow, Christian Gluud, Christoffer Hedetoft, Birger Thorsteinsson, Bianca Hemmingsen, Niels Wiinberg, Simone B. Sneppen, Søren S. Lund, Thure Krarup, Sten Madsbad, Thomas Almdal, Bendix Carstensen, Marit E. Jørgensen, the CIMT study group, T. Almdal, T. W. Boesgaard, L. Breum, B. Gade-Rasmussen, E. Duun, C. Gluud, C. Hedetoft, B. Hemmingsen, T. Jensen, T. Krarup, L. Lundby-Christensen, S. Lund, S. Madsbad, E. R. Mathiesen, O. Pedersen, H. Perrild, M. Røder, S. B. Sneppen, O. Snorgaard, L. Tarnow, B. Thorsteinsson, H. Vestergaard, A. Vaag, N. Wiinberg

**Affiliations:** 1 Steno Diabetes Center Copenhagen, A/S Niels Steensens Vej 2-4, 2820 Gentofte, Denmark; 2 Dept of Paediatrics, Nordsjaellands Hospital, Copenhagen University, Copenhagen, Denmark; 3 Department of Clinical Research, Nordsjaellands Hospital, Hillerød, Denmark; 4 Health, Aarhus University, Aarhus, Denmark; 5 Copenhagen Trial Unit, Centre for Clinical Intervention Research, Rigshospitalet, Copenhagen University Hospital, Copenhagen, Denmark; 6 Department of Medicine, University Hospital Køge, Køge, Denmark; 7 Department of Cardiology, Nephrology and Endocrinology, Nordsjællands University Hospital - Hillerød, Hillerød, Denmark; 8 Department of Clinical Physiology, Bispebjerg Hospital, University of Copenhagen, Copenhagen, Denmark; 9 Department of Medicine, Gentofte, Copenhagen University Hospital, Hellerup, Denmark; 10 Department of Endocrinology, Bispebjerg, Copenhagen University Hospital, Copenhagen, Denmark; 11 Department of Endocrinology, Hvidovre Hospital, University of Copenhagen, Hvidovre, Denmark; 12 Department of Clinical Medicine, Faculty of Health and Medical Sciences, University of Copenhagen, Copenhagen, Denmark; 13 Dept. of Endocrinology PE, Copenhagen University Hospital, Rigshospitalet, Denmark; 14 National Institute of Public Health, Southern Denmark University, Odense, Denmark

**Keywords:** Type 2 diabetes, Metformin, Autonomic neuropathy, Orthostatic blood pressure recovery, Complications, Peripheral neuropathy, Orthostatic hypotension, Cardiovascular autonomic neuropathy

## Abstract

**Background:**

Metformin has been shown to have both neuroprotective and neurodegenerative effects. The aim of this study was to investigate the effect of metformin in combination with insulin on cardiovascular autonomic neuropathy (CAN) and distal peripheral neuropathy (DPN) in individuals with type 2 diabetes (T2DM).

**Methods:**

The study is a sub-study of the CIMT trial, a randomized placebo-controlled trial with a 2 × 3 factorial design, where 412 patients with T2DM were randomized to 18 months of metformin or placebo in addition to open-labelled insulin. Outcomes were measures of CAN: Changes in heart rate response to deep breathing (beat-to-beat), orthostatic blood pressure (OBP) and heart rate and vibration detection threshold (VDT) as a marker DPN. Serum levels of vitamin B12 and methyl malonic acid (MMA) were analysed.

**Results:**

After 18 months early drop in OBP (30 s after standing) was increased in the metformin group compared to placebo: systolic blood pressure drop increased by 3.4 mmHg (95% CI 0.6; 6.2, p = 0.02) and diastolic blood pressure drop increased by 1.3 mmHg (95% CI 0.3; 2.6, p = 0.045) compared to placebo. Beat-to-beat variation decreased in the metformin group by 1.1 beats per minute (95% CI − 2.4; 0.2, p = 0.10). Metformin treatment did not affect VDT group difference − 0.33 V (95% CI − 1.99; 1.33, p = 0.39) or other outcomes. Changes in B12, MMA and HbA_1c_ did not confound the associations.

**Conclusions:**

Eighteen months of metformin treatment in combination with insulin compared with insulin alone increased early drop in OBP indicating an adverse effect of metformin on CAN independent of vitamin B12, MMA HbA_1c_.

*Trial registration* The protocol was approved by the Regional Committee on Biomedical Research Ethics (H–D-2007-112), the Danish Medicines Agency and registered with ClinicalTrials.gov (NCT00657943).

## Introduction

Patients with diabetes are at risk of neurological complications which can be manifested as cardiovascular autonomic neuropathy (CAN) and distal peripheral neuropathy (DPN). Both complications are highly prevalent with prevalence rates of CAN ranging from 20% in unselected diabetes populations [1, 2] to 65% in patients with long-standing diabetes [3]. Prevalence rates of DPN have been reported to vary from 10 to 41% [4, 5]. Both complications are associated with increased morbidity and mortality [6–11]. Reducing hyperglycaemia an integral part of the prevention for both CAN and DPN [12]. Due to efficacy and low price, metformin is recommended as the first-line drug in the treatment of type 2 diabetes worldwide [13]. Metformin may however potentially increase the risk of diabetic neurological complications, as metformin has been shown to impair absorption of vitamin B12 [14] leading to reduced levels of serum B12, which has been associated with both CAN [15] and DPN [16]. Conversely, metformin has also been shown to have neuroprotective effects and has been demonstrated to reduce pain sensation in patients with lumbar radicular pain [17] and in diabetic animal pain models [18], to protect against chemotherapy induced peripheral neuropathy [19], and to reduce apoptotic cell death in cortical neurons [20]. These beneficial effects have been suggested to be mediated by a metformin-induced reduction in oxidative stress [20]. Thus, it is unclear whether metformin may induce or prevent diabetic neuropathy.

To our knowledge no studies have assessed the consequences of treatment with metformin on neural function in a blinded randomised placebo-controlled design. The objective of this sub-study of the Copenhagen Insulin and Metformin Therapy (CIMT) trial [21–23] is to assess the effect of 18 months intervention with metformin versus placebo, each in combination with one of three different insulin analogue regimens on measures of CAN and DPN and to assess if vitamin B12 could mediate potential findings.

## Materials and methods

### Trial design

The present study is a sub-study of the CIMT trial. The CIMT trial [21–23] was an investigator initiated, multicentre, placebo-controlled superiority trial conducted from 2008 to 2012 at eight hospitals in the Capital Region of Denmark. The study was double-blinded for metformin/placebo treatment. The primary aim of the trial was to assess the effect of 18 months treatment with metformin 1 g twice daily versus placebo in combination with insulin on changes in carotid intima-media thickness in patients with type 2 diabetes [22]. Insulin treatment consisted of open label therapy with either 1: Biphasic insulin aspart before main meals one to three times daily or 2: Insulin aspart before main meals three times daily in combination with bedtime insulin detemir once daily or 3: Bedtime insulin detemir once daily. In total six different treatment groups.

All prior glucose lowering medications were discontinued at baseline. In total, 412 patients with type 2 diabetes (HbA_1c_ > 7.5% (≥ 58 mmol/mol) were enrolled in this 2x3 factorial designed study. Participants were randomly allocated into three groups: Patients with major cardiovascular events within the past 3 months, carotid artery stenosis > 70%, heart failure, recent cancer, renal or liver disease, alcohol or drug abuse, unstable retinopathy or pregnancy and patients breast feasting were not included. Insulin treatment was administered in a treat-to-target manner with a target of HbA_1c_ ≤ 7.0% (≤ 53 mmol/mol). In the original trial a total of 190 patients in the metformin group and 183 patients in the placebo group completed the trial and had usable outcome measures at baseline and follow-up. Among these, 15 patients in the metformin group and 28 patients in the placebo group discontinued treatment but were examined at end of the trial.

Participants were treated with antihypertensive agents and statins according to international guidelines at the time [24] and received aspirin 75 mg/d at the discretion of the investigators.

Study design and results of analyses on the primary outcome has been described previously [21–23]. The protocol was approved by the Regional Committee on Biomedical Research Ethics (H–D-2007-112), the Danish Medicines Agency and registered with ClinicalTrials.gov (NCT00657943).

Measures of CAN and DPN were performed at baseline and after 18 months follow-up.

### Objective measures of CAN

CAN was assessed by a standard orthostatic hypotension test and heart rate change when standing. After 10 min of supine rest the participant was asked to stand. Brachial blood pressure and heart rate was measured on the left arm at rest (two consecutive measures) and 30, 90 and 180 s after standing. Automated oscillometric blood pressure and heart rate recorders were used (AND UA-787plus, A&D medical, California, USA). Orthostatic hypotension was diagnosed if one or more of the following blood pressure changes were registered: systolic blood pressure drop ≥ 20 mmHg from baseline, diastolic blood pressure drop ≥ 10 mmHg from baseline or systolic blood pressure ≤ 90 mmHg according to the European Society of Cardiology [25]. CAN was additionally assessed by beat-to-beat analyses according to methods described by Ewing et al. [26]. Expiration/inspiration heart rate variability was assessed by 3-lead ECG traces measured during paced deep breathing at six breaths per minute in a supine position using a CardioFax 1150 (Nihon Kohden, Tokyo, Japan). Maximum and minimum heart rates during each breathing cycle were measured, and the means of the differences were calculated. Testing was done after 5 min of supine rest and resting heart rate was obtained prior to respiration testing.

### Objective measures of DPN

DPN was assessed by vibration detection threshold (VDT) measured by biothesiometry using a Bio-Thesiometer (Bio-medical instruments, Ohio, USA) by applying the biothesiometer on the distal tip of the first toe on each foot in turn and then increasing the amplitude until the patient indicates the sensation of vibration. To avoid the influence of asymmetrical and therefore possible non-diabetic neuropathies, only measures from the foot with the lowest vibration sensation threshold was used. Age-adjusted cut-off values of VDT [27] were used to diagnose DPN.

### Questionnaire based measures of autonomic neuropathy (AN) and DPN

Symptomatic AN and DPN was assessed by patient interviews. Symptomatic AN was considered present if the patient had experienced either faintness during shift in body position, faintness when upright for long durations, high resting heart rate, sweating when eating, early fullness, bloating or nausea when eating, urine retention, repetitive urinary infections or erectile dysfunction. Symptomatic DPN was considered present if the patient had experienced numbness of feet or legs, a burning or prickling sensation in feet or legs, increased sensibility to touch, inability to discriminate between hot and cold water when showering, muscle cramps in feet or legs or worsening of symptoms at night.

### Biochemical measures

Vitamin B12 (cobalamin) was measured by competitive immune analyses. Methyl malonic acid (MMA) was measured by gas chromatography–mass spectrometry. HbA_1c_ was measured by high performance liquid chromatography. HDL total and cholesterol were measured by standard enzymatic colorimetric techniques. Plasma creatinine was measures by two-point rate enzymatic technique and urinary albumin by quantitative immunological turbidimetry.

All analyses were done on a Vitros 5600 (Orhto Clinical Diagnostics, France) or a Roche Hitachi 912 (Rotkreuz, Switzerland) expect for HbA_1c_ which was analyzed on a Tosoh G7 (Tosoh Cooperation, Japan) and vitamin B12, which was analyzed on a Cobas e 601 (Rotkreuz, Switzerland). Serum LDL cholesterol was calculated using the Friedewald equation.

Vitamin B12 measures above 700 pmol/l were considered to be either due to faulty analyses, pathological states or excessive vitamin intake. Measures above this threshold were taken out of analyses.

### Anthropometric measures

Height and weight were measured with light indoor clothing, without shoes, using a fixed rigid stadiometer (Seca, Chino, USA) and an electronic scale (Mettler Toledo, Glostrup, Denmark), respectively.

### Statistical analyses

Patient characteristics are presented as means with standard deviations, or in cases of skewed distributions as median and interquartile ranges or as absolute numbers and percentages.

Analyses included only participants with complete data on the prespecified secondary outcomes addressed in the present study: blood pressure at rest and at 30 s, 90 s and 180 s after standing, the orthostatic hypotension diagnosis, heart rate variability during deep breathing (beat-to-beat), resting blood pressure, resting heart rate and VDT. The effect of treatment allocation (metformin and insulin or placebo and insulin) on outcomes at follow up was assessed by linear and logistic regression analyses adjusted for baseline values of the outcome in question (Model 1). Model 2 was additionally adjusted for treatment with metformin prior to the trial and change in vitamin B12 and MMA during the trial. A final level of adjustment (Model 3) also included change in HbA_1c_ during trial.

We tested the models for an interaction between metformin/placebo treatment and the three insulin regimens. Also, a modifying effect of metformin treatment prior to randomisation was tested. Model assumptions were assessed by residual plots. Between treatment group differences in outcome values at follow-up are shown as absolute values with 95% CI. The analyses were performed in an intention to treat approach including patients discontinuing treatment but examined at end of the trial.

Statistical analyses were performed in R version 3.5.2 (The R Foundation for Statistical Computing, http://www.R-project.org) and SAS version 9.4 (SAS Institute, Cary, NC, USA).

## Results

Anonymised aggregated data and a detailed account of all statistical analyses can be found at http://bendixcarstensen.com/SDC/CIMT/DOM/CIMT.pdf. For all analyses all model assumptions for the distribution of the model residuals were met.

### Study population

As described in detail previously [22] 206 participants were allocated to metformin treatment in combination with one of three insulin regimens. Similarly, 206 participants were allocated to placebo in combination with one of three insulin regimens. Not all patients had useable neuropathy measures at follow-up, which left 189 participants in the metformin group and 183 participants in the placebo group with neuropathy measures at both baseline and follow-up for analyses. Participants were predominantly male (68%), with a mean age of 61 years and a diabetes duration of 12,8 years. Symptoms of autonomic neuropathy and distal peripheral neuropathy were present in 16% and 38% of all participants, respectively. At baseline forty patients had orthostatic hypertension in the metformin group and thirty patients had orthostatic hypertension in the placebo group. Patient characteristics are shown in Table 1.

**Table 1:** Baseline characteristics of participants by allocation group

	Metformin + insulin (n = 183)	Placebo + insulin (n = 189)
Age (years)	61.0 (8.7)	60.3 (9.1)
Male, N (%)	140 (68)	141 (68)
Body mass index^a^	32.3 (4.2)	32.1 (4.2)
Smokers, N (%)	36 (18)	27 (13)
Median (IQR) alcohol consumption (units/week)	2 (0;6)	1 (0;5)
Duration of type 2 diabetes (years)	13.5 (6.2)	12.2 (6.5)
HbA1c (%)	8.6 (1.1)	8.5 (1.0)
HbA1c (mmol/mol)	70 (12)	69 (11)
LDL cholesterol (mmol/l)	2.2 (0.8)	2.2 (0.8)
eGFR^b^ (mL/min)	130 (44)	126 (45)
Vitamin B12 (pmol/l)	283 (200; 369)	275 (222; 359)
Methyl malonic acid (µmol/l)	0.20 (0.15; 0.25)	0.21 (0.16; 0.27)
Diabetic complications		
Symptomatic autonomous neuropathy N (%)	33 (16)	36 (18)
Symptomatic peripheral neuropathy N (%)	76 (37)	78 (38)
Prior cardiovascular disease N (%)^c^	45 (22)	55 (27)
Microalbuminuria N (%)	48 (24)	40 (20)
Macroalbuminuria N (%)	12 (6)	8 (4)
Simplex retinopathy N (%)	59 (30)	63 (31)
Proliferative retinopathy N (%)	15 (8)	10 (5)
Medication at baseline		
RAS blockade N (%)	159 (77)	149 (72)
Beta blocker N (%)	41 (10)	42 (10)
Acetylsalicylic acid	103 (54.5)	109 (59.6)
Diuretics N (%)	74 (18)	71 (17)
Statin N (%)	170 (83)	181 (88)

Values are means (SDs) unless stated otherwise. *IQR* interquartile range. ^a^Body mass index is calculated as weight (kg) divided by height (m)2. ^b^Calculated by the Cockcroft Gault equation: eGFR =  ( (140-age) × weight (kg) × constant)/serum creatinine (micromol/l), constant female: 1.04, male: 1.23. CVD, cardiovascular disease; eCCr, estimated creatinine clearance; ^c^Prior CVD was defined as one or more of the following: myocardial infarction, heart surgery, ischaemic heart disease, heart insufficiency, vascular surgery, stroke, transitory cerebral ischaemia, amputation *HbA1c* haemoglobin A1c, *HDL* high-density lipoprotein, *LDL* low-density lipoprotein, *RAS* Renin angiotensin system

### Blood pressure and CAN measures

Blood pressure and CAN measures in the metformin and placebo group are shown in Table 2. At inclusion, a total of 22% of the participants in the metformin group had orthostatic hypotension according to European Society of Cardiology guidelines compared to 16% in the placebo group (Table 2).

**Table 2:** Outcome variables and effect of treatment

	Outcomes at randomization		Outcomes at end of trial				
	Metformin + insulin	Placebo + insulin	Metformin + insulin	Placebo + insulin	Metformin vs placebo Model 1	Metformin vs placebo Model 2	Metformin vs placebo Model 3
Resting systolic blood pressure (mmHg)	136.5 (16.2)	134.5 (14.2)	136.7 (16.2)	134.5 (13.9)	1.06 (− 1.48; 3.59); P = 0.42	1.06 (− 1.59; 3.72); P = 0.43	0.87 (− 1.80; 3.56); P = 0.52
Resting diastolic blood pressure (mmHg)	80.8 (10.0)	79.9 (8.7)	78.7 (9.3)	78.7 (7.9)	− 0.37 (− 1.86; 1.11); P = 0.62	− 0.01 (− 1.52; 1.54); P = 0.99	0.04 (− 1.50; 1.58); P = 0.96
Systolic blood pressure 30 s after standing (mmHg)	130.4 (18.5)	128.8 (15.8)	128.7 (18.6)	130 (18.2)	3.09 (0.47; 5.71); P = 0.02	3.33 (0.58; 6.08); P = 0.02	3.35 (0.57; 6.12); P = 0.02
Diastolic blood pressure 30 s after standing (mmHg)	80.5 (10.7)	80.8 (9.1)	78.1 (10)	79.8 (9.7)	1.17 (− 0.05; 2.39); P = 0.06	1.38 (− 0.12; 2.64); P = 0.03	1.30 (0.30; 2.57); P = 0.045
Systolic blood pressure 90 s after standing (mmHg)	135.4 (17.9)	134.6 (15.3)	136.8 (17.8)	135.2 (17.3)	− 0.01 (− 2.28; 2.26); P = 0.99	0.44 (− 1.92; 2.80); P = 0.71	0.61 (− 1.77; 2.99); P = 0.62
Diastolic blood pressure 90 s after standing (mmHg)	83 (10.9)	83.3 (9.1)	81.3 (10.4)	82.1 (9.5)	0.44 (− 0.73; 1.60); P = 0.46	0.64 (− 0.58; 1.86); P = 0.30	0.62 (− 0.62; 1.86); P = 0.33
Systolic blood pressure 180 s after standing (mmHg)	135.8 (18.4)	135.4 (16.6)	135.8 (17.5)	135.2 (16.9)	1.10 (− 0.89; 3.10); P = 0.28	0.99 (− 1.07; 3.05); P = 0.34	0.97 (− 1.11; 3.05); P = 0.36
Diastolic blood pressure 180 s after standing (mmHg)	83.0 (10.9)	83.3 (9.1)	81.3 (10.4)	82.1 (9.5)	0.48 (− 0.64; 1.59); P = 0.40	0.45 (− 0.72; 1.62); P = 0.45	0.36 (− 0.82; 1.54); P = 0.55
Orthostatic hypotension diagnosis N (%)	41 (21.8)	30.0 (16.4)	49 (25.8)	38 (20.8)	1.24 (0.66; 2.31) P = 0.51	1.33 (0.70; 2.54); P = 0.38	1.33 (0.70; 2.54); P = 0.38
Beat-to-beat	9 (6; 16)	9 (6; 15)	8 (6; 14)	9 (6; 15)	− 1.14 (− 2.37; 0.09); P = 0.07	− 1.19 (− 2.47; 0.09); P = 0.07	− 1.09 (− 2.38; 0.20); P = 0.10
Resting heart rate (beats pr. minute)	70.1 (10.4)	69.3 (10.3)	70.6 (11.0)	69.0 (10.5)	1.03 (− 0.51; 2.57); P = 0.19	1.19 (− 0.40; 2.79); P = 0.14	1.17 (− 0.44; 2.78); P = 0.16
Heart rate 30 s after standing (beats pr. minute)	80.2 (12.3)	79.2 (11.1)	80.3 (12.8)	79.0 (11.7)	0.09 (− 1.18; 1.36); P = 0.89	0.18 (− 1.15; 1.51); P = 0.79	0.8 (− 1.26; 1.41); P = 0.91
Heart rate 90 s after standing (beats pr. minute)	78.2 (11.9)	79.2 (12.4)	78.8 (12.6)	77.6 (11.5)	0.19 (− 0.92; 1.30); P = 0.74	0.04 (− 1.12; 1.20); P = 0.94	0.04 (− 1.21; 1.39); P = 0.95
Heart rate 180 s after standing (beats pr. minute)	79.2 (12.6)	78.4 (11.75)	78.6 (13.0)	77.8 (11.2)	0.40 (− 0.66; 1.46); P = 0.46	0.19 (− 0.90 1.28); P = 0.73	0.02 (− 1.07 1.11); P = 0.97
Vibration detection threshold (volts)	20 (15; 29)	22 (16; 32)	22.5 (16; 30)	20 (16; 30)	− 0.33 (− 1.99; 1.32); P = 0.70	− 0.40 (− 2.11; 1.32); P = 0.65	− 0.49 (− 2.12; 1.25); P = 0.58
DPN Vibration detection threshold (age-adjusted) N (%)	25 (13.7)	23 (12.2)	34 (18.0)	26 (14.3)	1.42 (0.64; 13.17) P = 0.39.	1.33 (0.57; 3.08); P = 0.51	1.33 (0.57; 3.08); P = 0.51

Data are means (SD), medians (IQR) or numbers (%). Results of test for treatment effect are for continuous; outcomes linear regression estimates in absolute values (95% CL) and P values for group difference and for binary outcome; metformin associated odds ratios (95% CL) and P values for group difference. Models 1 adjusted for baseline values the outcome variable. Model 2 additionally adjusted for metformin treatment prior to trial and change in serum vitamin B12 and methyl malonic acid during trial. Model 3 additionally adjusted for change in HbA_1c_ during trial

**Fig. 1:**
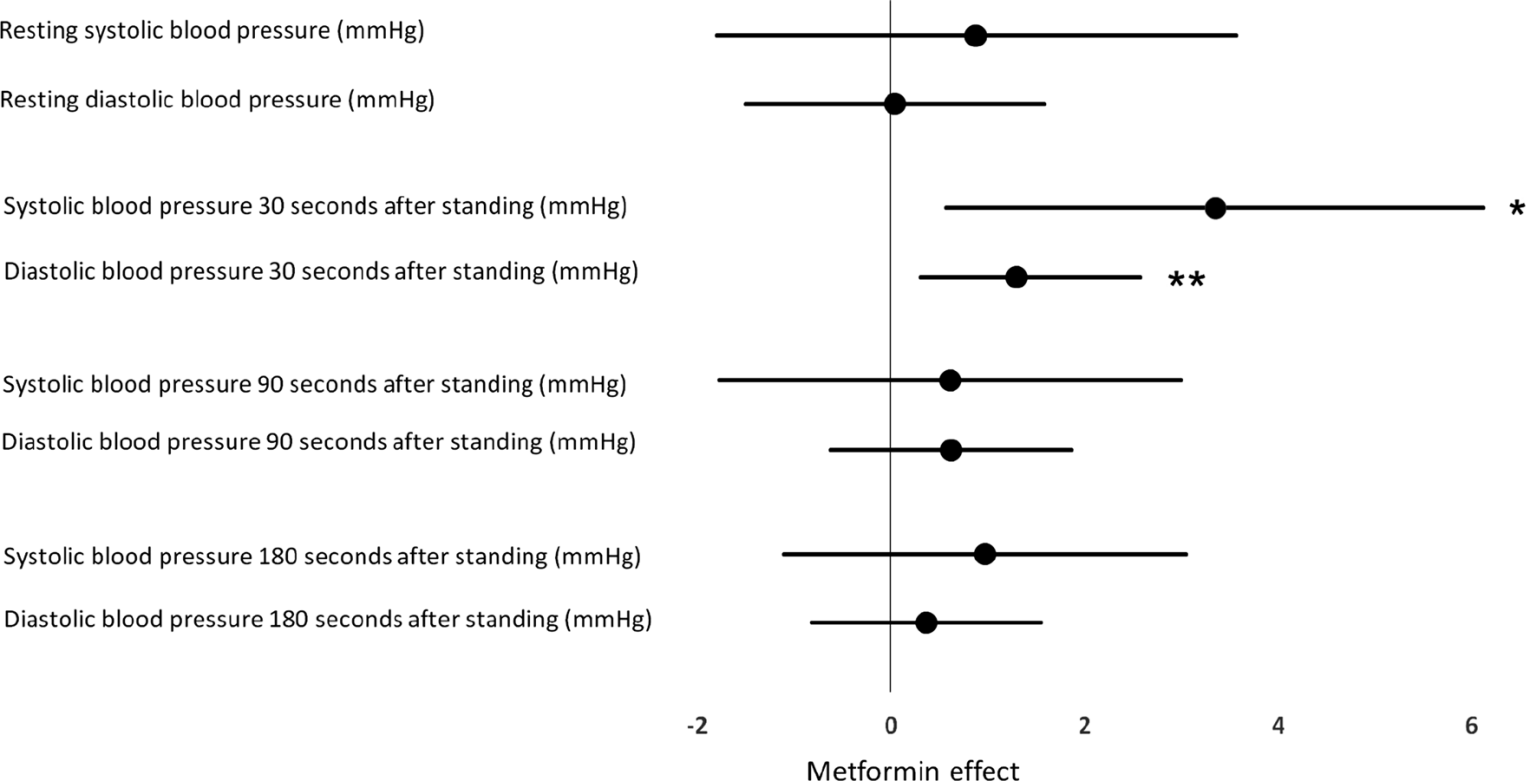
The effect of metformin on resting blood pressure and drop in blood pressure from resting in a supine position to standing. Estimates are adjusted for baseline values of outcomes, changes during trial in vitamin B12, methylmalonic acid ang HbA_1c_ and in addition pre-trial treatment of metformin. Effects are shown as mmHg and 95% CI. *p = 0.02. **p = 0.045 Only estimates for Model 3 are illustrated

After 18 months, a larger drop in blood pressure 30 s after standing was observed in the metformin group as compared to placebo. I models adjusted for changes in vitamin B12, MMA nd HbA_1c_ Systolic blood pressure drop increased by 3.4 mmHg (95% CI 0.6; 6.2, p = 0.02) and diastolic blood pressure-drop increased by 1.3 mmHg (95% CI 0.30; 2.6, p = 0.045) after 30 s standing. Estimates of blood pressure outcomes are shown in Table 2 and illustrated in Fig. 1. The odds ratio of progression to the diagnosis of orthostatic hypotension in the metformin group compared to placebo was 1.24 (0.66; 2.31) p = 0.51. Resting heart rate and change in heart rate as a response to standing was not associated with metformin treatment and no between group differences were detected.

Beat-to-beat variation decreased in the metformin group by 1.2 beats per minute (95% CI − 2.4; 0.2, p = 0.10).

No other significant effects of metformin treatment were on outcomes of CAN found. Estimates are shown in Table 2.

### Distal peripheral neuropathy measures

Measures of VDT were similar in both groups at baseline (Table 2). VDT increased in both groups during the trial and approximately 2.5% more patients had measures > 50 V at end of study (data not shown). No metformin effect was seen for continuous measures of VDT (Table 2) and the metformin associated odds ratio of progressing to a DPN during the trial was 1.42 (0.64;13.17) P = 0.39 (Table 2).

### Effect of vitamin B12 and methyl malonic acid

Seven patients (two in the metformin group and five in the placebo group) were excluded from analyses of vitamin B12 due to serum levels of vitamin B12 above 700 pmol/l.

During the trial vitamin B12 decreased in the metformin group by 19.9 pmol (95% Cl − 32.12; − 7.72, p < 0.01) and increased by 39.6 pmol/l (95% Cl 27.37;51.84, p < 0.01) in the placebo group (p < 0.01 for group difference).

Methyl malonic acid did not change in a clinically relevant magnitude during the trial. In the metformin group and the placebo group change during the trial was 0.00 µmol/l (95% Cl − 0.02; 0.01, p = 0.76) and 0.00 µmol/l (95% Cl − 0.02; 0.00, p = 0.01) in the respective groups. No group difference was seen (p = 0.17).

### Pre-trial metformin treatment and effect of insulin group allocation

When adjusting for metformin treatment prior to the trial and change in vitamin B12 and MMA during the trial, diastolic blood pressure-drop 30 s after standing was 1.4 mmHg (95% Cl 0.1; 2.6, p = 0.03) higher in the metformin group compared to placebo. No other estimates were significantly affected by adjustments (Table 2).

There were no interactions between insulin allocation group and effects of neither placebo nor metformin.

## Discussion

Our results showed that treatment of type 2 diabetes mellitus patients with 1-g metformin twice daily in combination with insulin significantly increases the initial systolic and diastolic blood pressure drop 30 s after standing when compared with placebo in combination with insulin treatment. The prevalence of orthostatic hypotension was approximately similar compared to studies in type 2 diabetes patients [28]. However, here was no difference in the progression toward the diagnosis of orthostatic hypotension nor was metformin treatment associated with peripheral neuropathy measures.

A numerical non-significant reduction in autonomic function was observed in the metformin group at end-off the trial where beat-to-beat variation were reduced compared with placebo. This trend was not explained by changes in vitamin B12 or MMA during the trial, indicating that metformin may affect autonomic and early orthostatic blood pressure response via other mechanisms. It is possible that detrimental effects on autonomic function may induce subclinical inflammation, which be part of the effect seen in the trial [29]. This effect of metformin on autonomic measures has not been described in diabetes patients previously. However, animal studies indicate that metformin may reduce resting blood pressure by a reduction of sympathetic autonomic tone [30, 31]. Animal studies consistently show a reduction of heart rate with metformin [30, 32, 33] also suggesting an effect on the autonomic nervous system. As stated, the effects of metformin on beat-to-beat measures were not statistically significant. Thus, other mechanisms may explain the hypotensive effect of metformin. It has been suggested that metformin cause a nitric-oxide dependent vasodilatation as seen in rats [34] and demonstrated in clinical research [35]. However, the present study could not reveal a metformin effect on resting blood pressure or heart rate. In addition, results from large randomised the trials in diabetes patients have shown neutral effects of metformin on blood pressure [36] suggesting that metformin does not affect resting blood pressure.

Another explanation of the findings of this study could be caused by a beneficial effect of the treatment in the control group rather than a detrimental effect of metformin. To reach the prespecified treatment goal of HbA_1c_ the placebo group in the CIMT trial was treated with significantly higher doses of insulin compared to the metformin group (1.36 IU/kg (95% CI 1.23 to 1.51) vs 1.04 IU/kg (95% CI 0.94 to 1.15), p < 0.001)), corresponding to approximately 36 units more insulin in the placebo group [22]. Insulin has been shown to have opposing vasodilator and vasoconstrictor actions although the net result of these attributes are a negligible effect on blood pressure [37]. Data from human studies show that insulin has neutral or detrimental effects on autonomic measures in humans [38, 39]. Hence it is unlikely that larger dose of insulin in the placebo group could cause a beneficial effect on blood pressure measures or autonomic function.

Furthermore, the placebo group gained significantly more weight during the trial compared to the metformin group (4.2 kg (95% CI 3.6 to 4.7) vs 1.6 kg (95% CI 1.1 to 2.1) p < 0.001) [22]. To our knowledge no studies have shown a positive effect of high weight or weight gain on orthostatic recovery or autonomic function. Conversely obesity is a risk factor for CAN [40] indicating that increased weight is unlikely to be protective of orthostatic hypotension or damaging to blood pressure recovery control. Further, the metformin group obtained a better degree of glycaemic control with a HbA1c of 7.97% compared with 8.27% in the placebo group. However, changes in HbA1c were not associated to outcomes especially autonomic outcomes as as seen in observational studies [41].The use of antihypertensive did not differ between the two groups before at trial start.

Pre-trial antidiabetic treatment may have played a confounding role in the analyses of outcomes. As described in the main outcome publications [22], 81–86% of participants were treated with metformin prior to enrolment. The effects of metformin described in the present study may be an effect of discontinuation of metformin at the start of the trial due to randomized allocation in the study groups. If this is the case, we would expect improvements in outcome measures in the placebo group and unchanged outcomes in the metformin group. Regarding systolic blood pressure 30 s after rising a numeric increase is seen in the placebo group and a numeric decrease in the metformin group, indicating a beneficial effect of metformin discontinuation. In any case, metformin seems to have an effect on outcome measures. Adjusting for pre-trial metformin treatment did not affect outcomes (results available in the on-line data report).

Given the effect of metformin is only seen in the early phase of blood pressure recovery, it is possible that the effect may primarily affect mechanisms related to initial orthostatic hypotension (IOH). IOH is defined as a transient and significant blood pressure drop within 30 s after standing [25]. The pathological mechanism causing IOH are not fully elucidated but may be the result of several adverse acute reactions to standing [42]; local elevated blood flow in legs due to muscle contraction induced arterial-venous blood pressure gradient mismatch, peripheral vasodilatation and systemic sympathetic withdrawal mediated by a mechanoreceptor response. If indeed metformin is associated with IOH, the most plausible link would be through the latter mechanism, as muscle pump disorders or isolated local vasomotor dysfunction associated with metformin is unlikely, thus suggesting a primary effect on the autonomic nervous system.

Metformin can cause a measurable reduction in vitamin B12 within months [43]. It is possible that minor reductions in serum vitamin B12 can be associated with CAN as it has been demonstrated earlier that diabetes patients in the lower range of a near-normal serum vitamin B12 have affected measures of CAN [15]. In the present study changes in serum levels of vitamin B12 and MMA did not confound associations between metformin treatment and outcome measures, indicating that the effect of metformin may be due to other mechanisms than changes in vitamin B12.

Whether changes in orthostatic blood pressure recovery caused by Metformin are reversible remains unclear. An possible autonomic component of these changes may be reversible as autonomic dysfunction may be reversible in some cases [44, 45].

In relation to DPN our results are uniform. No effects of metformin were seen.

### Strengths and limitations

#### Strengths

This study in the first double-blinded (for metformin/placebo treatment) placebo-controlled randomised trial with 18 months follow-up on the effect of metformin treatment on measures of CAN in a large population of type 2 diabetes patients.

### Limitations

Measures of autonomic function used in the present study are crude. No direct measures of sympathetic function e.g. plasma epinephrine and norepinephrine, microneurography or sudomotor function were applied. The beat-to-beat variation mainly reflects parasympathetic function [46], while orthostatic hypotension is mainly caused by a dysfunctional sympathetic nervous system [47], the aforementioned beat-to-beat measure may not be a valid measure to link autonomic dysfunction to reduced orthostatic recovery. To distinguish between initial orthostatic hypotension and “classical” orthostatic hypotension continuous blood pressure measurements during the orthostatic hypotension test procedure are needed [25]. In the present study it is therefore not possible to distinguish between these two forms of orthostatic hypotension. In addition, no standardised questionnaires were used to assess symptoms of neuropathy reducing the validity of theses outcomes.

## Conclusion

Our results indicate that metformin treatment in combination with insulin may influence early orthostatic blood pressure response as metformin treatment attenuated orthostatic blood pressure 30 s after standing. These adverse changes could be mediated by changes in autonomic function.

Changes in serum levels of vitamin B12, methyl malonic acid and HbA_1c_ did not confound our findings. As only few autonomic measured were found to be associated with metformin treatment and in a non-statically significant manner it is premature to conclude that metformin indeed has a detrimental effect on nerve function. Future studies with a primary focus on the effect of metformin on diabetic cardiovascular autonomic neuropathy are needed to confirm our findings and to elucidate the underlying pathological mechanisms before conclusions on this possible metformin-induced adverse effect can be drawn.

## Data Availability

The datasets used and/or analysed during the current study are available from the corresponding author on reasonable request.

## Abbreviations

CAN: Cardiovascular autonomic neuropathy
DPN: Distal peripheral neuropathy
VDT: Vibration detection threshold
CIMT trial: Copenhagen Insulin and Metformin Therapy trial

## References

[1] Valensi P, Paries J, Attali JR (2003). Cardiac autonomic neuropathy in diabetic patients: influence of diabetes duration, obesity, and microangiopathic complications–the French multicenter study. Metabolism..

[2] Ziegler D, Dannehl K, Muhlen H, Spüler M, Gries FA (1992). Prevalence of cardiovascular autonomic dysfunction assessed by spectral analysis, vector analysis, and standard tests of heart rate variation and blood pressure responses at various stages of diabetic neuropathy. Diabetic Med.

[3] Low PA, Benrud-Larson LM, Sletten DM (2004). Autonomic symptoms and diabetic neuropathy: a population-based study. Diabetes Care.

[4] Martin CL, Albers JW, Pop-Busui R, Group DER (2014). Neuropathy and related findings in the diabetes control and complications trial/epidemiology of diabetes interventions and complications study. Diabetes Care.

[5] Tesfaye S, Stevens LK, Stephenson JM (1996). Prevalence of diabetic peripheral neuropathy and its relation to glycaemic control and potential risk factors: the EURODIAB IDDM Complications Study. Diabetologia.

[6] Wheeler SG, Ahroni JH, Boyko EJ (2002). Prospective study of autonomic neuropathy as a predictor of mortality in patients with diabetes. Diabetes Res Clin Pract.

[7] Gerritsen J, Dekker JM, TenVoorde BJ (2001). Impaired autonomic function is associated with increased mortality, especially in subjects with diabetes, hypertension, or a history of cardiovascular disease: the Hoorn Study. Diabetes Care.

[8] Vinik AI, Ziegler D (2007). Diabetic cardiovascular autonomic neuropathy. Circulation.

[9] Crawford F, Inkster M, Kleijnen J, Fahey T (2007). Predicting foot ulcers in patients with diabetes: a systematic review and meta-analysis. QJM.

[10] Young LH, Wackers FJ, Chyun DA (2009). Cardiac outcomes after screening for asymptomatic coronary artery disease in patients with type 2 diabetes: the DIAD study: a randomized controlled trial. JAMA.

[11] Yun JS, Park YM, Cha SA, Ahn YB, Ko SH (2018). Progression of cardiovascular autonomic neuropathy and cardiovascular disease in type 2 diabetes. Cardiovasc Diabetol..

[12] Boulton AJ, Vinik AI, Arezzo JC (2005). Diabetic neuropathies: a statement by the American Diabetes Association. Diabetes Care.

[13] Davies MJ, D’Alessio DA, Fradkin J (2019). Correction to: Management of hyperglycaemia in type 2 diabetes, 2018. A consensus report by the American Diabetes Association (ADA) and the European Association for the Study of Diabetes (EASD). Diabetologia.

[14] Reinstatler L, Qi YP, Williamson RS, Garn JV, Oakley GP (2012). Association of biochemical B (1) (2) deficiency with metformin therapy and vitamin B (1) (2) supplements: the National Health and Nutrition Examination Survey, 1999-2006. Diabetes Care.

[15] Hansen CS, Jensen JS, Ridderstrale M, Vistisen D, Jorgensen ME, Fleischer J. Vitamin B12 deficiency is associated with cardiovascular autonomic neuropathy in patients with type 2 diabetes. Journal of diabetes and its complications. 2016:. 10.1016/j.jdiacomp.2016.08.025 27638143

[16] Aroda VR, Edelstein SL, Goldberg RB (2016). Long-term Metformin Use and Vitamin B12 Deficiency in the Diabetes Prevention Program Outcomes Study. J Clin Endocrinol Metab.

[17] Taylor A, Westveld AH, Szkudlinska M (2013). The use of metformin is associated with decreased lumbar radiculopathy pain. Journal of pain research..

[18] Ma J, Yu H, Liu J, Chen Y, Wang Q, Xiang L (2015). Metformin attenuates hyperalgesia and allodynia in rats with painful diabetic neuropathy induced by streptozotocin. Eur J Pharmacol.

[19] Mao-Ying QL, Kavelaars A, Krukowski K (2014). The anti-diabetic drug metformin protects against chemotherapy-induced peripheral neuropathy in a mouse model. PLoS ONE.

[20] El-Mir MY, Detaille D, Villanueva G (2008). Neuroprotective role of antidiabetic drug metformin against apoptotic cell death in primary cortical neurons. J Mol Neurosci.

[21] Lundby Christensen L, Almdal T, Boesgaard T (2009). Study rationale and design of the CIMT trial: the Copenhagen Insulin and Metformin Therapy trial. Diabetes Obes Metab.

[22] Lundby-Christensen L, Tarnow L, Boesgaard TW (2016). Metformin versus placebo in combination with insulin analogues in patients with type 2 diabetes mellitus-the randomised, blinded Copenhagen Insulin and Metformin Therapy (CIMT) trial. BMJ Open.

[23] Lundby-Christensen L, Vaag A, Tarnow L (2016). Effects of biphasic, basal-bolus or basal insulin analogue treatments on carotid intima-media thickness in patients with type 2 diabetes mellitus: the randomised Copenhagen Insulin and Metformin Therapy (CIMT) trial. BMJ Open.

[24] Standards of medical care in diabetes–2008. Diabetes Care. 2008; 31 Suppl 1: S12–54. 10.2337/dc08-S012 18165335

[25] Moya A, Sutton R, Ammirati F (2009). Guidelines for the diagnosis and management of syncope (version 2009). Eur Heart J.

[26] Ewing DJ, Clarke BF (1982). Diagnosis and management of diabetic autonomic neuropathy. Br Med J.

[27] Bloom S, Till S, Sonksen P, Smith S (1984). Use of a biothesiometer to measure individual vibration thresholds and their variation in 519 non-diabetic subjects. Br Med J.

[28] Gannon J, Claffey P, Laird E, Newman L, Kenny RA, Briggs R (2020). The cross-sectional association between diabetes and orthostatic hypotension in community-dwelling older people. Diabet Med.

[29] Bhati P, Alam R, Moiz JA, Hussain ME (2019). Subclinical inflammation and endothelial dysfunction are linked to cardiac autonomic neuropathy in type 2 diabetes. Journal of diabetes and metabolic disorders..

[30] Muntzel MS, Abe A, Petersen JS (1997). Effects of adrenergic, cholinergic and ganglionic blockade on acute depressor responses to metformin in spontaneously hypertensive rats. The Journal of pharmacology and experimental therapeutics..

[31] Petersen JS, Liu W, Kapusta DR, Varner KJ (1997). Metformin inhibits ganglionic neurotransmission in renal nerves. Hypertension.

[32] Muntzel MS, Hamidou I, Barrett S (1999). Metformin attenuates salt-induced hypertension in spontaneously hypertensive rats. Hypertension.

[33] Peuler JD, Miller JA, Bourghli M, Zammam HY, Soltis EE, Sowers JR (1997). Disparate effects of antidiabetic drugs on arterial contraction. Metabolism..

[34] Katakam PV, Ujhelyi MR, Hoenig M, Miller AW (2000). Metformin improves vascular function in insulin-resistant rats. Hypertension.

[35] Mather KJ, Verma S, Anderson TJ (2001). Improved endothelial function with metformin in type 2 diabetes mellitus. J Am Coll Cardiol.

[36] Wulffele MG, Kooy A, de Zeeuw D, Stehouwer CD, Gansevoort RT (2004). The effect of metformin on blood pressure, plasma cholesterol and triglycerides in type 2 diabetes mellitus: a systematic review. J Intern Med.

[37] Muniyappa R, Montagnani M, Koh KK, Quon MJ (2007). Cardiovascular actions of insulin. Endocr Rev.

[38] Paolisso G, Manzella D, Rizzo MR (2000). Effects of insulin on the cardiac autonomic nervous system in insulin-resistant states. Clin Sci (Lond)..

[39] Charles LE, Andrew ME, Sarkisian K (2014). Associations between insulin and heart rate variability in police officers. Am J Human Biol.

[40] Spallone V, Ziegler D, Freeman R (2011). Cardiovascular autonomic neuropathy in diabetes: clinical impact, assessment, diagnosis, and management. Diabetes Metab Res Rev.

[41] Matsutani D, Sakamoto M, Minato S (2018). Visit-to-visit HbA1c variability is inversely related to baroreflex sensitivity independently of HbA1c value in type 2 diabetes. Cardiovasc Diabetol..

[42] Wieling W, Krediet CT, van Dijk N, Linzer M, Tschakovsky ME (2007). Initial orthostatic hypotension: review of a forgotten condition. Clin Sci (Lond)..

[43] Wulffele MG, Kooy A, Lehert P (2003). Effects of short-term treatment with metformin on serum concentrations of homocysteine, folate and vitamin B12 in type 2 diabetes mellitus: a randomized, placebo-controlled trial. J Intern Med.

[44] Andersen ST, Witte DR, Fleischer J (2018). Risk factors for the presence and progression of cardiovascular autonomic neuropathy in type 2 diabetes: ADDITION-Denmark. Diabetes Care.

[45] Jun JE, Lee SE, Choi MS, Park SW, Hwang YC, Kim JH (2019). Clinical factors associated with the recovery of cardiovascular autonomic neuropathy in patients with type 2 diabetes mellitus. Cardiovasc Diabetol..

[46] Vinik AI, Maser RE, Mitchell BD, Freeman R (2003). Diabetic autonomic neuropathy. Diabetes Care.

[47] Perlmuter LC, Sarda G, Casavant V, Mosnaim AD (2013). A review of the etiology, associated comorbidities, and treatment of orthostatic hypotension. Am J Ther.

